# Iliopsoas fibrosis after revision of total hip arthroplasty revealed by ^68^Ga-FAPI PET/CT: a case report

**DOI:** 10.3389/fmed.2024.1328630

**Published:** 2024-02-19

**Authors:** Yiqun Wang, Yabing Sun, Junlei Song, Xiaojun Zhang, La Li, Zhihui Shen, Jiahe Tian, Yingfang Ao

**Affiliations:** ^1^Department of Sports Medicine, Peking University Third Hospital, Institute of Sports Medicine of Peking University, Beijing Key Laboratory of Sports Injuries, Beijing, China; ^2^Department of Nuclear Medicine, The First Medical Centre, Chinese PLA General Hospital, Beijing, China; ^3^Department of Orthopedics, The First Medical Centre, Chinese PLA General Hospital, Beijing, China

**Keywords:** 68Ga-FAPI, PET/CT, THA, skeletal muscle, fibrosis

## Abstract

**Background:**

Total hip arthroplasty (THA) is a well-established surgical procedure that has been extensively validated to alleviate pain, enhance joint function, improve the ability to perform daily activities, and enhance overall quality of life. However, this procedure is associated with certain complications, among which skeletal muscle fibrosis is a frequently overlooked but significant complication that can lead to persistent pain. Currently, there is no effective method for diagnosing skeletal muscle fibrosis following total hip arthroplasty.

**Case report:**

We report a 75-year-old male patient who complained of left groin pain after revision total hip arthroplasty. Serological examinations, X-rays, and bone scan results were all normal. However, during the ^68^Ga-FAPI PET/CT examination, we observed significant radiotracer uptake along the iliopsoas muscle. This abnormal uptake pattern suggested potential biological activity in this specific area. Combined with physical examination, the patient was diagnosed with iliopsoas fibrosis.

**Conclusions:**

The presented images indicated that the uptake pattern was an important indicator for diagnosis, and the prospect of fibroblast activation protein in the diagnosis of skeletal muscle fibrosis has shown certain application value.

## Introduction

Total hip arthroplasty (THA) is a well-established surgical procedure known for enhancing quality of life, alleviating pain and restoring joint function. Nevertheless, complications can arise, with reported incidence ranging from 0 to 38.3% (1, 2). Skeletal muscle fibrosis, unlike more common complications such as periprosthetic joint infection or dislocation, often goes unnoticed. In clinical practice, ultrasonography and magnetic resonance imaging (MRI) are the more commonly used methods of examining muscles (3, 4). Ultrasound is cost-effective, easily accessible, and simple to implement, and offers the advantages of no radiation exposure and relatively few contraindications. Modern ultrasound systems have ideal soft tissue resolution; however, they lack standardized criteria for assessing muscle repair and can be subject to operator bias. MRI has specific sequences for detecting muscle edema and fat replacement, and provides quantitative methods and standardization (5, 6). However, it is time-consuming and noisy, and may be less effective in patients with metallic implants. Additionally, anatomical imaging studies may reveal non-specific structural changes that could affect the examination accuracy.

With the emergence of ^68^Ga-fibroblast activation protein inhibitor (^68^Ga-FAPI) (7, 8), its applications have broadened beyond oncology to encompass various fields, including autoimmune diseases, infections, and more (9, 10). Previous studies have shown that in normal tissues, fibroblast activation protein (FAP) is expressed at lower levels, while activated fibroblasts and fibrotic tissues express elevated levels of FAP (11, 12). In our preclinical research, we observed that in a muscle injury model, the expression of FAP gradually decreased in the normal repair model, while in the fibrotic model, FAP continued to be expressed. Theoretically, ^68^Ga-FAPI holds potential for diagnosing skeletal muscle fibrosis. In this report, we present a case of iliopsoas fibrosis diagnosis utilizing ^68^Ga-FAPI following THA.

## Case report

**FIGURE 1:**
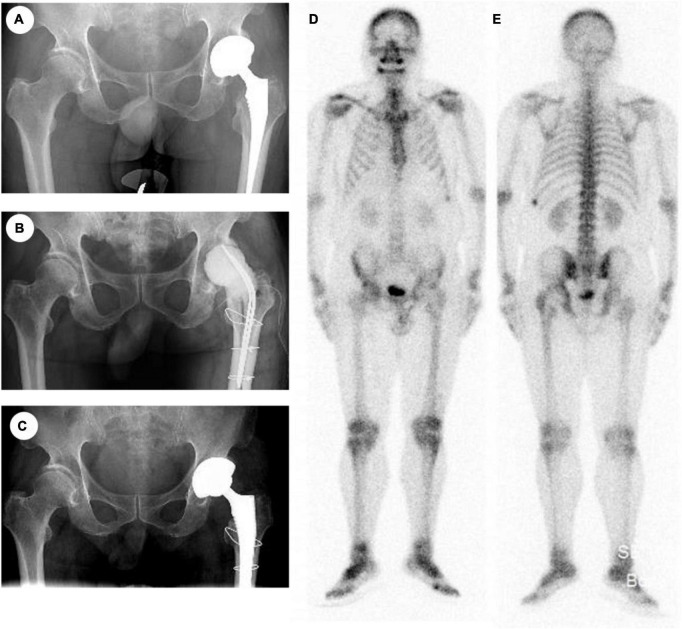
**(A)** X-ray of primary operation. **(B)** X-ray of two-stage reconstruction. **(C)** X-ray after revision. **(D,E)** Bone scan.

A 75-year-old man presented to our hospital 3°years ago complained about pain in the left THA for approximately 6°months (Figure 1A). Five years prior, he had undergone primary THA due to a femoral neck fracture, and post-surgery, he was diagnosed with periprosthetic joint infection (PJI) based on serological tests and synovial fluid culture. A two-stage reconstruction approach was selected, and the patient received antibacterial-loaded bone cement treatment for 3 months (Figure 1B) before undergoing revision surgery. One year after the revision surgery, the patient complained of groin pain. Bone scans (Figures 1D, E) showed no abnormal uptake in the left THA, while X-rays (Figure 1C) and serological results (WBC: 5.39 109/L, CRP: 1.81 mg/dl, ESR: 10°mm/h, IL-6: 1.5°pg/ml) indicated no apparent abnormalities.

To further confirm the diagnosis, ^68^Ga-FAPI PET/CT scan was conducted (this test was approved by the Ethics Committee of our hospital and registered at the Chinese Trial Registry: ChiCTR2000041204, and written informed consent was obtained from the patient for the purpose of scientific research). ^68^Ga-FAPI was synthesized as previously outlined (13). Imaging involved partial body scans from the pelvis to the knee, using a time-of-flight PET/CT scanner (uMI510; United Imaging Healthcare, Shanghai, China). Patients underwent scans 1 h after injection radiopharmaceuticals. Each bed position had a 4-minute acquisition time. Low-dose CT (120 kV, 30–50 mA) provided anatomical localization and attenuation correction. The reconstruction employed a standard ordered-subset expectation–maximization algorithm. Two junior nuclear medicine physicians independently calculated the SUVmax and it was considered feasible that the results of the SUVmax were consistent.

**FIGURE 2:**
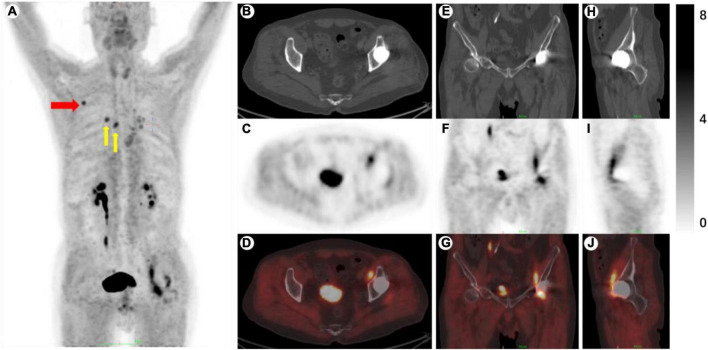
**(A)** MIP. **(B–D)** Axial images of CT, PET, and fused. **(E–G)** Coronal images. **(H–J)** Sagittal images. Red arrow, right scapular spine, SUVmax: 4.9. Yellow arrow, multiple calcified lymph nodes, SUVmax: 4.5.

^68^Ga-FAPI PET/CT revealed increased uptake in the right scapular spine (Figure 2A, red arrow, SUVmax: 4.9), and multiple calcified lymph nodes with enhanced uptake in the lung hilum and mediastinum (Figure 2A, yellow arrow, SUVmax: 4.5). Additionally, increased uptake was observed along the iliopsoas around the left hip joint, with an SUVmax of 6.3 (Figures 2A–J). During the physical examination, the patient was positioned supine and tested for hip flexion, demonstrating a muscle strength of grade 4 on the left side. Subsequently, with the patient placed prone, overextension of the hip joint induced significant pain and restricted range of motion (less than 10 degrees). Drawing on existing literature regarding FAP’s role in fibrosis and extracellular matrix (9, 14), the diagnosis of iliopsoas fibrosis was made for this case. Considering the softening and dispersing effect of ultrasound therapy on fibrous tissue, ultrasound therapy combined with dynamic stretching was employed for this patient. In the follow-up at 6 months, the patient reported no pain, and the strength of the hip flexors had reached a level of 5 (normal range), with a hip extension angle of 15 degrees.

## Discussion

The occurrence of muscle fibrosis results from the complex interplay of various factors, including tissue injury, chronic inflammation, autoimmune reactions, and genetic changes. It is characterized by the excessive deposition of extracellular matrix components (15, 16).

Typically, muscle fibrosis is diagnosed through tissue biopsies, but this method is invasive, subject to sampling variability, and provides limited spatial information. To address these limitations, multiple imaging tools and techniques have been explored for the diagnosis of muscle fibrosis. Among these methods, ultrasound and MRI are relatively common diagnostic approaches. Ultrasound offers cost-effectiveness, ease of access, and straightforward deployment. It does not involve radiation and generally has few contraindications for most patients. Modern ultrasound systems provide high-resolution soft tissue imaging. However, there is currently no unified standard for assessing muscle repair using ultrasound, and results may be influenced by the operator’s technical proficiency.

MRI has specific sequences for detecting muscle edema and fat replacement, along with quantitative methods and standardization. However, MRI examinations often require a longer duration, can be noisy, and may yield suboptimal results for patients with metallic implants. Furthermore, anatomy-based imaging studies may be susceptible to interference from non-specific anatomical changes, affecting diagnostic accuracy (17).

For the diagnostic evaluation of skeletal muscle fibrosis, imaging studies that provide only anatomical information have been somewhat underwhelming. The ability to capture protein and molecular-level changes and whether it can serve as a tool for staging, monitoring, or even screening has become the next hot research direction. Positron Emission Tomography (PET) is a molecular imaging technique that seamlessly integrates the visualization of anatomy studies with the high sensitivity and specificity of molecular examinations, making it one of the representative examinations in precision medicine (17).

Kimura et al. (18) conducted imaging of iliopsoas tendinitis patients using CT, MRI, and ^18^F-fluorodeoxyglucose (^18^F-FDG). The assessment of CT or MRI was hindered by artifacts produced by metal prosthetics. The use of ^18^F-FDG revealed metabolic activity along the iliopsoas muscle, confirming the presence of iliopsoas tendinitis. This indicates that ^18^F-FDG can detect lesions earlier and more precisely than conventional examinations.

However, ^18^F-FDG imaging relies on glucose metabolism, which has relatively poor specificity (19–21). In our previous research, non-specific uptake in areas like the intestines and blood vessels reduced the efficiency of image analysis (22). Therefore, the use of more specific radiotracers is worth exploring in the assessment of skeletal muscle injury repair.

FAP is not expressed in normal tissues and has been hailed as the next billion-dollar nuclear theranostics target due to its ideal sensitivity and specificity (23), and both other researchers’ studies and our team (10, 24–26) showed that FAPI had the advantages of both sensitivity and specificity. In our previous study (10), we used ^68^Ga-FAPI to differentiate aseptic loosening from periprosthetic joint infection. However, we also noticed that in addition to loosening and infection, there were other conditions that caused pain after THA, such as osteolysis, bursitis of the greater trochanter, and pseudotumor (26). It made us realize that although SUVmax is an important indicator, uptake pattern also plays an important role in diagnosis, which could not only delineate the range of lesions but also distinguish different etiologies. In this case, patients who had undergone revision surgery and continued to experience discomfort, with no apparent abnormalities observed in routine examinations, and due to the presence of metallic implants, underwent ^68^Ga-FAPI PET/CT. Through nuclear medicine imaging, the patient not only received an accurate diagnosis but also achieved a comprehensive presentation of the lesion.

However, our study had several limitations. First, ultrasound, MRI, and ^18^F-FDG examinations were not conducted in this case. Then, this study only included one case and requires validation with a larger sample size. Finally, there was no in-depth investigation into the underlying mechanisms. These limitations will be addressed in future studies.

## Conclusions

Skeletal muscle fibrosis is a not uncommon but often overlooked complication of THA, and there is a lack of effective methods for its diagnosis. Through the presentation of this case and the discussion of the role of FAP in fibrosis, ^68^Ga-FAPI PET/CT holds a theoretical advantage in the diagnosis of muscle fibrosis. The specific mechanisms and broader clinical research in this field deserve further exploration.

## Data availability statement

The original contributions presented in the study are included in the article/Supplementary material, further inquiries can be directed to the corresponding author.

## Ethics statement

The studies involving humans were approved by the Ethics Committee of Chinese PLA General Hospital. The studies were conducted in accordance with the local legislation and institutional requirements. The participants provided their written informed consent to participate in this study. Written informed consent was obtained from the individual(s) for the publication of any potentially identifiable images or data included in this article.

## Author contributions

YW: Funding acquisition, Writing – original draft. YS: Conceptualization, Writing – review & editing. XZ: Software, Writing – review & editing. LL: Writing – review & editing. JS: Methodology, Writing – original draft. ZS: Project administration, Writing – review & editing. JT: Methodology, Supervision, Writing – review & editing. YA: Methodology, Supervision, Validation, Writing – review & editing.
